# Mass Spectrometry of Single GABAergic Somatic Motorneurons Identifies a Novel Inhibitory Peptide, As-NLP-22, in the Nematode ***Ascaris suum***

**DOI:** 10.1007/s13361-015-1177-z

**Published:** 2015-07-15

**Authors:** Christopher J. Konop, Jennifer J. Knickelbine, Molly S. Sygulla, Colin D. Wruck, Martha M. Vestling, Antony O. W. Stretton

**Affiliations:** Department of Zoology, University of Wisconsin-Madison, Madison, WI 53706 USA; Parasitology and Vector Biology Training Program, University of Wisconsin-Madison, Madison, WI 53706 USA; Department of Chemistry, University of Wisconsin-Madison, Madison, WI 53706 USA; Neuroscience Training Program, University of Wisconsin-Madison, Madison, WI 53706 USA

**Keywords:** MALDI-TOF/TOF, Single-cell MS, in situ Hybridization, Neuropeptides, Nematode

## Abstract

Neuromodulators have become an increasingly important component of functional circuits, dramatically changing the properties of both neurons and synapses to affect behavior. To explore the role of neuropeptides in *Ascaris suum* behavior, we devised an improved method for cleanly dissecting single motorneuronal cell bodies from the many other cell processes and hypodermal tissue in the ventral nerve cord. We determined their peptide content using matrix-assisted laser desorption/ionization time-of-flight (MALDI-TOF) mass spectrometry (MS). The reduced complexity of the peptide mixture greatly aided the detection of peptides; peptide levels were sufficient to permit sequencing by tandem MS from single cells. Inhibitory motorneurons, known to be GABAergic, contain a novel neuropeptide, As-NLP-22 (SLASGRWGLRPamide). From this sequence and information from the *A. suum* expressed sequence tag (EST) database, we cloned the transcript (*As-nlp-22*) and synthesized a riboprobe for in situ hybridization, which labeled the inhibitory motorneurons; this validates the integrity of the dissection method, showing that the peptides detected originate from the cells themselves and not from adhering processes from other cells (e.g., synaptic terminals). Synthetic As-NLP-22 has potent inhibitory activity on acetylcholine-induced muscle contraction as well as on basal muscle tone. Both of these effects are dose-dependent: the inhibitory effect on ACh contraction has an IC_50_ of 8.3 × 10^–9^ M. When injected into whole worms, As-NLP-22 produces a dose-dependent inhibition of locomotory movements and, at higher levels, complete paralysis. These experiments demonstrate the utility of MALDI TOF/TOF MS in identifying novel neuromodulators at the single-cell level.

**Electronic supplementary material:**

The online version of this article (doi:10.1007/s13361-015-1177-z) contains supplementary material, which is available to authorized users.

**Graphical Abstract:**
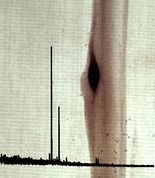
ᅟ

## Introduction

In the mid-20th century, there was a resurgence in interest in simple nervous systems, typically found in invertebrates, where the numbers of neurons are low [1, 2]. The hope was that the low numbers would allow anatomical descriptions of the individual neurons and their synaptic connections to be combined with electrophysiological measurements to give a “functional wiring diagram,” and that this would give us an understanding of the way the nervous system controls behavior. Progress has been slow: it is now recognized that the simplicity of these nervous systems is more apparent than real [3]. A large part of the complexity of even these “simple nervous systems” comes from the existence of a large number of modulating signaling molecules, typically peptides and amines, which affect the neurons and synapses in subtle and complicated ways, each of which needs to be worked out. The description of the functional wiring diagram was incomplete because it did not include the role of these neuromodulators.

The inadequacy of the naïve “functional wiring diagram” approach and the importance of the role of neuropeptides and other modulators in affecting circuit properties have been particularly well described in the stomatogastric ganglion (STG) of Crustacea [4–12]. In this ganglion, the cell numbers are small (N = 30) and despite the complicated morphology of the neurons, their synaptic connectivity has been determined, both anatomically and physiologically [11, 12]. However, enormous complexity is introduced by the large number of modulators that reach STG neurons from neurons in other ganglia [7, 13–16]. These modulators can dramatically change the properties of the neurons and synapses in the STG, and each one may produce complex and different responses on different targets, reconfiguring the network into different functional circuits. This is the important concept of “circuit switching” [12, 17, 18]. The analysis of the system is ongoing as more modulators are discovered [4–7, 19, 20].

Our attempt to simplify the analysis of circuitry has taken advantage of the properties of the nervous system of the nematode *Ascaris suum*. Like that of other nematodes, its nervous system is extremely simple, both numerically (adult female *A. suum* has a total of 298 neurons [21]; the *Caenorhabditis elegans* hermaphrodite has 302 neurons [22]) and morphologically (typical neurons have no more than two branch points); yet they have an extensive array of neuropeptides [23–29]. In order to understand a particular neuronal circuit at the cellular level, the salient peptides must be identified, and their biological activities analyzed. These are crucial parts of the structure–function description of a circuit that are needed to create a meaningful functional wiring diagram. Similarly, the identification of the neurons that express each neuropeptide (i.e., the cellular expression pattern), is functionally important.

We are concentrating on one aspect of the behavior of *A. suum*, the control of locomotion. *A. suum* exhibits a simple locomotory behavior characterized by a propagating two-dimensional waveform produced by alternating contractions and relaxations of dorsal and ventral muscles; these muscles are controlled by serial sets of dorsal and ventral excitatory and inhibitory motorneurons distributed along the length of the body [30]. The cell bodies of these motorneurons are relatively large and are found in a highly reproducible ordered array in the ventral cord; this has allowed for extensive biochemical and physiological characterization of these neurons [30–37]. After locating the classic neurotransmitters in individual motorneurons and investigating their function, we have taken several paths to identifying endogenous peptides. Initially, we used chemical isolation by multi-step high pressure liquid chromatography (HPLC), followed by the use of specific antibodies and in situ hybridization [27, 28, 38, 39]. More recently, we have used mass spectrometry (MS), first on isolated ganglia and then on single identified neurons [23, 40, 41], and have identified and sequenced over 100 peptides from *A. suum*; this is a fraction of the over 250 putative peptides that are predicted [29, 40, 42]. Already it is clear that most of the neuropeptides that have been tested affect the physiological properties of the motorneurons and/or muscle cells, although many of these peptides are expressed in neurons that are not an obvious part of the locomotory circuitry. We have now improved the dissection techniques so that we can isolate individual identified motorneurons from the ventral nerve cord, and isolate and sequence their endogenous neuropeptides.

As in other organisms, peptides in nematodes are generated from precursor proteins by proteolysis and other post-translational processing steps [29, 43]. Nematode peptides and genes are highly conserved within the phylum and many are grouped into three large families, originally described in *C. elegans* [44–46]: the peptides in two of these families, FMRFamide-like peptides (*flps*) and insulin-like peptides (*ins*), are sequence-related within their respective families. A third family consists of a more diverse group of neuropeptide-like proteins (*nlps*); in this group, there are subfamilies of related peptides, but the subfamilies have little sequence similarity to each other [29, 44].

In order to identify the endogenous peptides of motorneurons, we have dissected single motorneurons and analyzed them by single-cell mass spectrometry. This was made possible by a necessary refinement, described in this paper, of the single cell dissection technique previously developed in *A. suum* [23, 41], and used in conjunction with matrix-assisted laser desorption/ionization time-of-flight (MALDI-TOF) MS. The quantities of peptide present in single cells are sufficient not only to identify individual peptides in individual identified neurons by their molecular mass but also to determine their sequence by tandem MS (MS/MS). We confirm the de novo sequence determination by tandem MS of synthetic peptide, showing that the fragmentation pattern is the same. Determination of the peptide sequence by MS is what leads this work; the sequence is enabling, since it permits searching of nucleic acid databases for the encoding transcripts, which can be used for the synthesis of riboprobes for in situ hybridization; it also is essential for making synthetic peptide for functional studies, since the quantities of peptide readily available from biological material are not sufficient (unless heroic purifications are carried out).

We report here the identification, localization, and bioactivity of a peptide expressed in the GABAergic VI and DI inhibitory ventral cord motorneurons. This peptide, As-NLP-22 (SLASGRWGLRPamide), is encoded by the *A. suum* neuropeptide-like protein gene *As-nlp-22*, and is cleaved from the precursor protein encoded by this gene. We have confirmed the cellular expression of this gene by in situ hybridization; this is an important validation of the adequacy of the single neuron dissection technique, since it shows that the mass spectra are not massively contaminated by peptides present in processes (e.g., synaptic terminals) from neighboring neurons. Pharmacological experiments with synthetic As-NLP-22 show that it is a potent inhibitor of acetylcholine (ACh)-induced contraction of *A. suum* muscle, and that it induces paralysis in intact behaving worms.

A search of nematode expressed sequence tag (EST) libraries for As-NLP-22-like peptides identified predicted homologous peptides in several parasitic and free-living species, including *C. elegans*. Because of its inhibitory effects on muscle and its apparent ubiquity among nematodes, it promises to be an excellent candidate for the development of novel anthelminthic drugs.

## Materials and Methods

### Animals

Live *A. suum* were collected from pig intestines at a slaughterhouse and maintained in phosphate-buffered saline (PBS, 140 mM sodium chloride, 10 mM sodium phosphate, pH 6.8–7.5) at 37°C. The PBS was changed daily and worms were used within 3 d of collection.

### Sample Preparation for Mass Spectrometry

Adult female *A. suum* were injected with 0.1–0.3 mL of 2 mg/mL collagenase (Sigma Blend H; Sigma, St Louis, MO, USA) in *Ascaris* saline [4 mM sodium chloride, 125 mM sodium acetate, 24.5 mM potassium chloride, 5.9 mM calcium chloride, 4.9 mM magnesium chloride, 5 mM 3-(*N*-morpholino) propanesulfonic acid (MOPS) buffer, pH 6.8] and incubated for 1.5–2 h at 37°C to dissociate the muscle tissue. A 6–7 cm portion of the worm anterior to the gonopore was removed and transferred to a Sylgard-lined dish. After cutting longitudinally between the dorsal nerve cord and the right lateral line, the preparation was pinned open, the lips were cut off, and the pharynx was removed. The preparations were rinsed three times with 170 mM ammonium acetate before the isotonic glycerol dissecting solution was added (170 mM ammonium acetate in 30% glycerol [47–50]). Dissecting needles made from electrolytically-sharpened tungsten wire were used to isolate a desired neuron from the many neuronal processes and hypodermis in which it is embedded. For better visualization, some preparations were bathed in 0.8 mM Methylene Blue in 170 mM ammonium acetate for 20–30 s, then cleared with 170 mM ammonium acetate, and dissected as described above.

Isolated cells were transferred to a Bruker MTP stainless steel MALDI target (Bruker Daltonics, Billerica, MA, USA). Cells were cleared with 0.5 μL isopropanol [51] to remove the glycerol and any remaining extracellular salts. Individual neurons were spotted with 60–100 nL of saturated α-cyano-4-hydroxycinnamic acid (CHCA; Sigma) in 70% acetonitrile (ACN; Fisher Scientific, Waltham, MA, USA), 1% trifluoroacetic acid (TFA; Sigma), or saturated 2,5-dihydroxybenzoic acid (DHB; Sigma) in 1:1 HPLC grade methanol and water (Fisher Scientific) using a Nanoliter Cool Wave Syringe II [52].

### Mass Acquisition

A Bruker Ultraflex III MALDI-TOF/TOF MS (Bruker Daltonics) equipped with a Smartbeam laser and LIFT-TM cell was used to obtain MS and MS/MS spectra with Bruker Compass v. 1.2 software. Because the cells contain relatively small quantities of peptide, all spectra were obtained from 50 laser shots per acquisition. MS spectra were obtained in positive ion reflectron mode, with an *m/z* range of 500–4000 Da. Instrument settings were optimized for maximum detection sensitivity using the Bruker Flex Control software: ion source 1 voltage, 25.0 kV; ion source 2 voltage, 20.5 kV; reflector 1 voltage, 26.5 kV; reflector 2 voltage, 14.5 kV; lens voltage, 9.5 kV. Synthetic peptide standards were not suitable for calibration of the instrument because the laser intensity needed for analysis of single cells was often far too high when analyzing pure standard, making it impossible to identify the monoisotopic peak of the ion needed for accurate calibration. Therefore, a reference spectrum from a freshly dissected *A. suum* AVK neuron, with known peptide peaks [23], was used for external calibration of the instrument.

### Acetylation of Peptides

Following deposition of the cell onto the target surface, 0.5 μL of isopropanol was applied to each cell to wash away the glycerol solution, followed by 0.5 μL of methanol/ acetic anhydride (3:1). The cells were allowed to dry before each wash. Cells were then covered with matrix as described above.

### Oxidation of Peptides

In cells exposed to Methylene Blue, methionine residues were partially oxidized to the sulfoxide, with a mass shift of +16 Da, and tryptophan residues gave rise to +16 and +32 adducts [41].

### Assignment of Peaks and Interpretation of Mass Spectra

Spectra were analyzed using Bruker Daltonics flex-Analysis 3.0 software. The software automatically assigned masses to peaks in each MS spectrum. Each MS/MS spectrum underwent background subtraction and smoothing before the automatic assignment of masses. In some cases, peak *m/z* values were added manually. Peaks were considered significant if they were twice the intensity of the baseline noise.

Molecular masses and ion fragmentation patterns were calculated by Protein Prospector MS-Product (http://prospector.ucsf.edu). Spectra containing peaks with *m/z* values corresponding to masses ±0.2 Da of known *A. suum* peptides were temporarily assigned. Confirmation of the assignments was carried out by MS/MS and chemical modifications to the peptides.

De novo sequencing of unidentified peaks was carried out by hand. To establish the b series (N-terminal) and y series (C-terminal) ions for each MS/MS spectrum, candidate b_2_ and y_1_ ions were chosen as a starting point for sequencing. To grow each ion series, the annotation function in the flex-Analysis software was used to calculate the distance between adjacent peaks to identify each amino acid until the full sequence was deduced in both directions. The sequence was entered into Protein MS-Prospector and the spectrum was analyzed for the complete set of sequence ions, including internal fragments and immonium ions. Verification of peptide sequence was carried out by comparing the experimental MS/MS spectrum with that of synthetic peptide. Further verification was carried out by comparing sequences to tBLASTn searches and cloning of *A. suum* gene sequences. Raw spectral image files were transferred to Adobe Illustrator (San Jose, CA, USA) for annotation.

### Database Searches

Database searches were conducted using methods described in recent publications [23, 41, 42]. Briefly, all predicted peptide sequence assignments were searched using tBLASTn (National Center for Biotechnology Information [NCBI], Bethesda, MD, USA; http://www.ncbi.nlm.nih.gov/BLAST/) against *A. suum* ESTs and a library of 447,546 genomic survey sequences (GSS). For all searches, program settings were modified for searching short sequences using a word size of 2, an E value of 20,000, and a PAM30 matrix. Search results were translated using the ExPASy Translate tool (http://au.expasy.org/tools/dna.html) and examined for putative peptide cleavage sites, and then for C-terminal glycine for C-terminal amidation of peptides. Genomic sequences were analyzed for mRNA splice sites directly by PCR cloning of the mRNA sequences and compared with genomic sequences [53]. Signal peptides were predicted using SignalP 4.0 (http://www.cbs.dtu.dk/services/SignalP/).

### Peptide Synthesis

Peptides were synthesized (Fmoc chemistry) by the University of Wisconsin-Madison Biotechnology Center (UWBC). The integrity of the synthesis was monitored by MALDI-TOF MS and HPLC.

### RNA Isolation and cDNA Preparation

Twenty-thirty *A. suum* heads were flash-frozen in liquid nitrogen and ground to a fine powder. Total RNA was isolated using a Nucleospin Nucleic Acid Purification Kit (Clontech, Mountain View, CA, USA). First strand cDNA was generated for PCR using a Superscript First Strand Synthesis System for RT-PCR (Life Technologies, Grand Island, NY, USA). Rapid amplification of cDNA ends (RACE)-ready cDNA for 5′- and 3′-RACE was created from total RNA using a SMARTer RACE cDNA amplification kit (Clontech) according to the manufacturer’s instructions.

### Transcript Identification by PCR and RACE

To obtain information about the transcript that encoded this peptide, primers were designed for PCR and RACE from sequences in the *A. suum* EST library (accession no. BI593877). RACE reactions were performed using an Advantage 2 PCR Kit (Clontech) according to the manufacturer’s instructions with the 5′RACE primer nlp22-5RACE3 (5′-GTGCACACGATCCACAAAGCCTTCGAAA-3′) and 3′RACE primer nlp22-3RACE (5′-CAAAACGCTCCTTAGCTAGCGGTCGTTG-3′). The PCR reaction was performed using the forward primer nlp22-3RACE2 (5′-GCTCGTTGCTTGCGGTGCTCTTCGTTTCGAT-3′) and the reverse primer nlp22-5RACE (5′-CCCATCGAGGCCAAGTTCGTCAGTATAAATCG-3′) in a 50 μL reaction as follows: 2 μL of *A. suum* cDNA or 2 μL of cDNA reaction solution with no reverse transcriptase as a control, 5 μL of 10X PCR Gold buffer (Applied Biosystems, Foster City, CA, USA), 2-8 μL of 25 mM MgCl_2_, 1 μL each of 10 mM dNTPs, 1 μL of each primer (20 μM), and 0.25 μL of AmpliTaq Gold Polymerase (Applied Biosystems; 5 U/μL). The PCR conditions were programmed into the Eppendorf Mastercycler Gradient as follows: 95°C for 3 min, followed by 40 cycles of 94°C for 30 s, 66°C or 68°C for 30 s, 72°C for 1 min.

PCR and RACE products were run on a 1% agarose gel, and novel bands were excised and purified using a Qiaquick Gel Extraction Kit (Qiagen, Chatsworth, CA, USA). The purified products were cloned into *E. coli* using a TOPO TA Cloning Kit (Invitrogen, Carlsbad, CA, USA), and the plasmid DNA was isolated with a Qiagen Miniprep Kit. Automated sequencing was carried out by the DNA Sequencing Facility at the UWBC. Sequence electropherograms were viewed on Chromas Lite software (Technelysium Pty. Ltd., South Brisbane, Australia). Sequences were analyzed using the ExPASy Translate Tool and T-Coffee Multiple Sequence Alignment Tool (Swiss Institute of Bioinformatics, Lausanne, Switzerland), and signal peptide sequences were identified using SignalP 4.0.

### Riboprobe Synthesis

The *As-nlp-22* specific riboprobe was created using primers *nlp22-*3RACE2 and *nlp22*-5RACE for PCR as described above. Products were cloned and sequenced to confirm the fidelity of the sequence and to determine the orientation of the insert in the vector. The target sequences of the riboprobe are shown in Figure 3b. The constructs were linearized using restriction enzymes NotI and SpeI (New England Biolabs, Beverly, MA, USA). Linearized plasmids were used as a template to synthesize an antisense (experimental) and a sense (negative control) digoxigenin-labeled riboprobe (Maxiscript SP6/T7 kit; Ambion, Austin, TX, USA; digoxigenin-11-dUTP; Roche Applied Science, Indianapolis, IN, USA) as previously described [54]. To remove unincorporated nucleotides, the reactions were run through NucAway Spin Columns (Ambion). Probe integrity and concentration were determined by gel electrophoresis with Sybr Gold staining (Molecular Probes, Eugene, OR, USA) and nylon membrane dot blots (Roche Applied Science protocol).

### Localization of As-nlp-22 by In Situ Hybridization

*A. suum* whole mount preparations were prepared by injecting large female worms with 0.1–0.3 mL of 2 mg/mL collagenase in autoclaved *Ascaris* saline and incubating in a beaker of PBS at 37°C for 1.5–2 h. The anterior 6 cm of the worm was taken and rinsed in autoclaved 100 mM phosphate buffer (pH 7.4) and the heads were transferred to a Sylgard-coated dissecting dish. The heads were cut longitudinally between the dorsal nerve cord and the right lateral line, the lips were removed, and the heads were pinned flat. Any remaining muscle cells were gently removed with forceps. The preparations were rinsed several times in 100 mM phosphate buffer and fixed in 1% paraformaldehyde in 100 mM phosphate buffer (pH 7.4) on a rocking table at 4°C overnight.

After fixation, the head preparations were processed as previously described [54], with the following modifications: incubation in proteinase K solution was increased to 30 min at 37°C, and staining was allowed to occur for up to 16 h in the dark, or until staining of the cells was evident and background staining began to occur. The staining reaction was stopped with Milli-Q water, and the heads were rinsed with Milli-Q water (2 × 10 min). The preparations were mounted in Clear-Mount (Electron Microscopy Sciences, Hatfield, PA, USA) and allowed to dry. Microphotographs were taken with a Zeiss AxioCam MRc camera on a Zeiss Universal microscope.

### Characterization of As-NLP-22 Bioactivity in Muscle Strips

Strips of dorsal muscle were obtained by cutting large female *A. suum* longitudinally along the lateral lines and isolating a 2 cm strip approximately 1 cm posterior to the head. Each preparation contained dorsal muscle cells (all longitudinal) and the motor axons of the dorsal nerve cord. The ends of the preparation were tied with silk thread. One end was attached to a fixed hook in a 7 mL chamber containing normal *Ascaris* saline stirred by bubbling nitrogen gas, and the other end was tied to a FORT25 force transducer (World Precision Instruments, Sarasota, FL, USA). The output of the transducer was routed through a TBM4M transbridge (World Precision Instruments) and recorded on a computer using Data-Trax or LabScribe2 software (World Precision Instruments), and the data were analyzed for tension/time relationships.

Baseline contractions were measured by adding 5 μM ACh to the chamber by micropipette, followed by rinses of *Ascaris* saline. The preparation was then exposed to 10 μM peptide solution in *Ascaris* saline for 10 min. The chamber was rinsed, fresh peptide was added, and ACh-induced contraction was measured by adding 5 μM ACh immediately after exposure to peptide solution, followed by rinses with peptide-free *Ascaris* saline. Muscle strip contraction in response to ACh was measured at 10, 20, 30, 40, and 50 min after exposure to peptide, with rinses of peptide-free *Ascaris* saline between doses of ACh. For comparison, control muscle strips were subjected to ACh-induced contraction in peptide-free *Ascaris* saline at the same time points. Responses of the muscle strips were reported as a percentage of the maximum ACh contraction observed for each muscle strip. A dose–response curve was created by repeating the procedure described above with peptide concentrations of 10 μM, 1 μM, 100, 10, 1, 0.1, 0.01 nM and comparing the results to control muscle strips.

### Statistical Analysis

Contractile responses and baseline tension were reported as the mean ± SE for each peptide concentration and displayed in Prism 6 software (GraphPad, San Diego, CA, USA). Statistical significance between peptide and control trials was determined using unpaired *t*-tests, with significance levels set at *P* < 0.05 or *P* < 0.01. The IC_50_ was calculated using the four-parameter logistic (4PL) curve and Prism 6 software, with constants estimated by nonlinear regression.

### Characterization of NLP Effects in Intact Worms

To examine the broader behavioral responses associated with neuropeptides, we used the whole worm injection assay [55]. Worms used in this assay are allowed to move freely inside an 18-mm-diameter × 41-cm-long glass tube filled with PBS at 37°C and closed off with rubber stoppers. This tube is similar in diameter to the porcine small intestine, which is the natural habitat of these worms. The worms were allowed to acclimate to the tube for 5 min, followed by 5 min of observation prior to injection to establish a baseline for locomotory activity. Then, the worms were partially removed from the tube and injected with 0.1 mL of 10 μM peptide solution in *Ascaris* saline, and placed back inside the tube. Handling of the worms was kept to a minimum. Locomotory activity was observed for 60 min post-injection, with recorded observations grouped into 5-min increments. Digital photos were captured with a Nikon D50 camera at 5 min pre-injection, immediately before and immediately after injection, and at 5-min intervals post-injection.

### Multiple Alignments

BLAST searches of the nematode EST library were performed using both As-NLP-22 and Ce-NLP-22 as queries. Selected sequences were imported into MEGA5.1 [56] for alignment by MUSCLE [57]. The alignments were imported into Jalview [58] for display.

## Results and Discussion

### Anatomical Background

**Figure 1:**
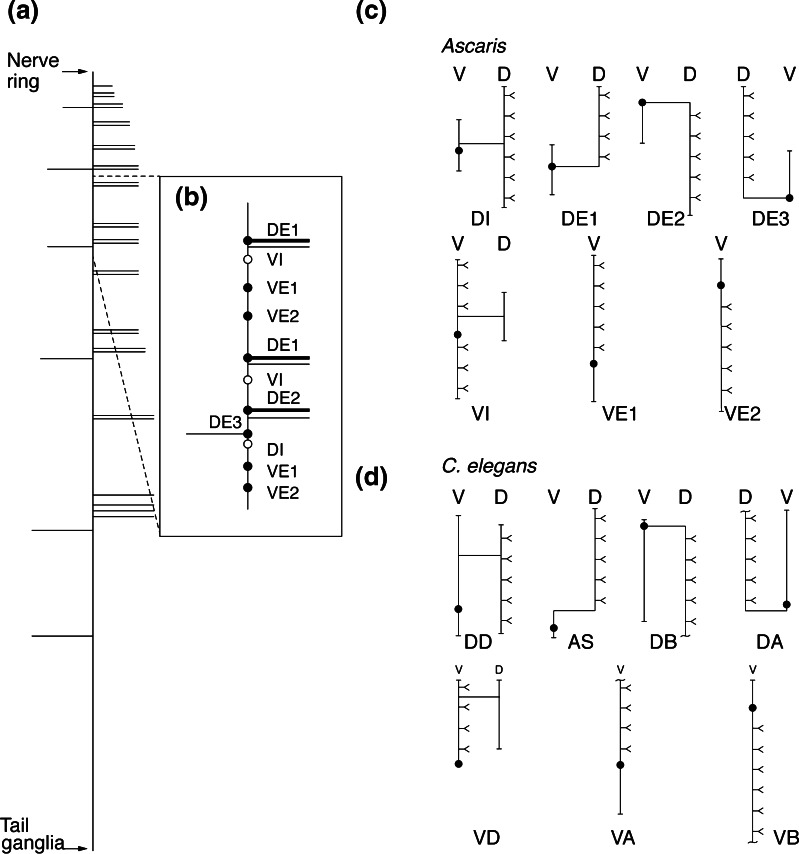
Schematic of the ventral cord motorneurons of *A. suum*. **(a)** Diagram of the ventral nerve cord adapted from Stretton et al. (1978) showing the position and handedness of the motorneuron commissures. The ventral nerve cord is represented by the vertical line. Right- and left-handed commissures leave the cord in an ordered array, indicated by the horizontal lines. Arrows show the positions of the nerve ring and tail ganglia. Some of the commissures at the anterior end of the ventral cord have been omitted for clarity. **(b)** Stereotypical pattern of motorneuron cell bodies found in each segment (segments 2–5) and their associated commissures. This figure shows the third segment. Filled circles indicate cell bodies of excitatory motorneurons. Open circles indicate cell bodies of inhibitory motorneurons. DE1 and DE2 commissures have a larger diameter than the inhibitory commissures with which they are paired [36]. **(c)**, **(d)** Schematic of the seven types of **(c)** *A. suum*, and **(d)** *C. elegans* segmental motorneurons. Filled circles indicate cell bodies, all found in the ventral nerve cord (V). Vertical lines emanating from the cell body represent the ventral nerve cord processes, and horizontal lines represent commissures that connect dorsal (D) and ventral processes. Forked projections represent axonal synapses onto muscle

The basic morphologic and physiological properties of adult *A. suum* motorneurons have been well described [30], as has their classic neurotransmitter phenotype [36, 37]. The somatic motorneurons, which innervate the dorsal and ventral musculature, comprise seven morphologic and physiological types, four (DE1, DE2, DE3, and DI) controlling dorsal muscle, and three (VE1, VE2, and VI) controlling ventral muscle (Figure 1). The cell bodies of all these neurons are located in the ventral nerve cord, and dorsal muscle is innervated via neural processes (commissures) that extend from the ventral to the dorsal nerve cord. These seven types of motorneurons are analogous to seven classes of motorneurons found in *C. elegans* [22, 30]. The dorsal and ventral excitors (DE and VE) are cholinergic [36] and the corresponding inhibitors (DI and VI) are GABAergic [34, 37]. These motorneurons occur in repeating patterns along the length of the worm, such that each of the five “segments” includes 11 motorneurons, four of which (DE1, VE1, VE2, and VI) are present in two copies, with only a single copy of DE2, DE3, and DI [30].

Cell identification relies on several features (see Figure 1a, b). First, the DE1, DE2, and DE3 neurons have commissures that emerge from the cell body; in the case of the DE1 and DE2 neurons, the identification is reinforced by the diameter of the commissure, which is larger than that of the accompanying commissure from an inhibitory neuron [36]. Second, the cell body of the VI neuron is the next posterior cell body to the DE1/VI commissure pair. The cell body of the DI motorneuron is posterior to the DE2/DI commissure pair; the relative position of the DE3 and DI cell bodies is variable, but DE3 is recognized by the commissure emerging from its cell body. Five of these types (DE1, DE2, DE3, DI, and VI) have been investigated electrophysiologically, and their properties are unusual [59]. None of these neurons conducts action potentials; instead they have high membrane resistance, which enables them to signal passively over long distances. They have long processes (axons) within the nerve cords, and synapse onto muscle cells along the length of the axon, providing synaptic input to muscle cells over a distance of several centimeters [32].

### Dissection of Single Motorneurons

Initially, the dissection of single motorneurons from the ventral nerve cord was difficult because of the close association with hypodermis and the other processes within the nerve cords. The use of an isotonic dissecting solution containing glycerol has permitted the isolation of all the cell bodies of the motorneurons in any chosen length of the ventral nerve cord with minimal contamination from surrounding tissue [47]. The mass spectra of different individual cells are highly distinctive, but they fall into classes that correspond to morphologic and physiological subtypes. The peptides present are few in number, and individual peptides can readily be isolated as single peaks for subsequent sequencing by tandem MS. Once the sequence has been established, the peptide can be synthesized and tested for its bioactivity. Sequence information also enables searches of EST databases for the encoding transcript, which can then be cloned and used to design and synthesize gene-specific mRNA probes for in situ hybridization (ISH). In all cases, ISH has confirmed the expression of the peptide-encoding transcript in the identified motorneuron that contains the peptide itself. ISH also determines the expression of the transcript in other neurons throughout the entire nervous system.

### Peptide Characterization by MS of Single Inhibitory Ventral Cord Motorneurons

**Figure 2:**
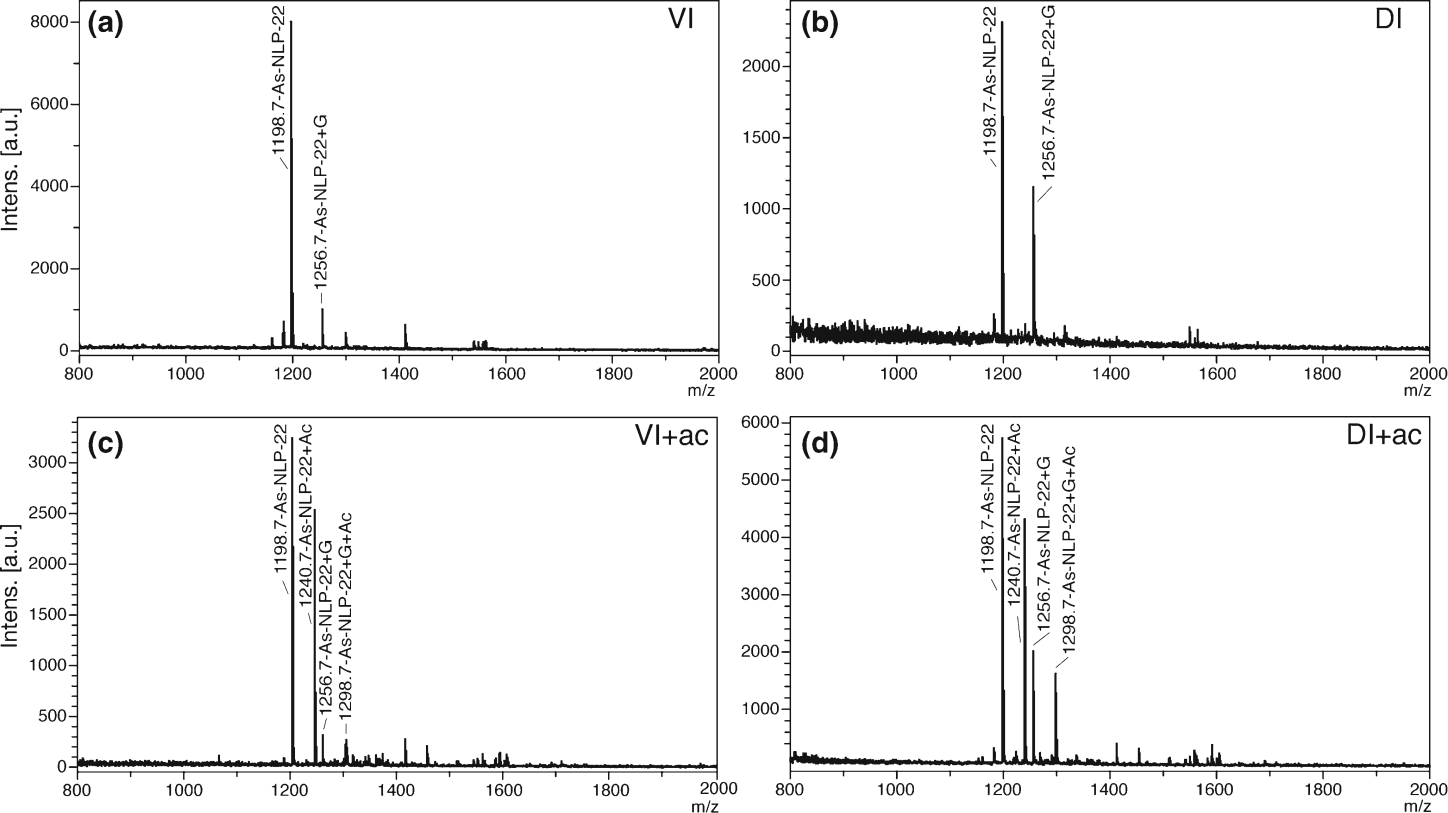
Neuropeptides from single ventral cord inhibitory motorneurons. **(a)**, **(b)** Mass spectra from individual **(a)** VI and **(b)** DI neurons. **(c)**, **(d)** Spectra from individual **(c)** VI and **(d)** DI neurons treated with acetic anhydride each causing a +42 Da mass shift. In these peptides, only the N-terminal α-amino group is substituted

From the ventral cord (VC), the cell bodies of individual VI and DI neurons were dissected from the anterior three segments of the worm (Figure 1a, b) and analyzed by MALDI-TOF MS for their peptide content. Spectra from VI and DI were virtually identical, each containing only two intense to moderate peaks, which is relatively few compared with other cell types analyzed in previous studies [23, 41]. All spectra from both VI and DI neurons contained an intense peak with *m/z* of 1198.7. In 11/17 VI and 12/15 DI spectra, there were also smaller peaks with *m/z* 1256.7 (Figure 2a, b). Interestingly, direct tissue analysis by MS on a dissected portion of the *A. suum* ventral nerve cord, which included ventral cord inhibitory motorneuron cell bodies and their neurites, indicated the presence of both peaks, among many others, but they had not been previously sequenced [40].

In MALDI-TOF MS, certain on-target chemical modifications are straightforward, and can identify functional groups in peptides. For example, in some preparations, cells were briefly stained with Methylene Blue to aid in cell identification during dissection (see Materials and Methods). Exposure to Methylene Blue has been shown to partially oxidize methionine residues to the sulfoxide, causing a +16 shift in *m/z*; to a lesser extent, tryptophan residues may be oxidized to give +16 Da or +32 Da adducts [41, 60]. In three of 17 VI spectra and one of 15 DI spectra we observed a small peak with an *m/z* of 1214.7 (1198.7 + 16) and another at 1230.7 (1198.7 + 2×16) (data not shown). These minor peaks suggested the presence of a tryptophan residue. On-target acetylation produced a mass shift of +42 Da for both the 1198.7 and 1256.7 peaks, consistent with the presence of a single amino group in each peptide (Figure 2c, d).

**Figure 3:**
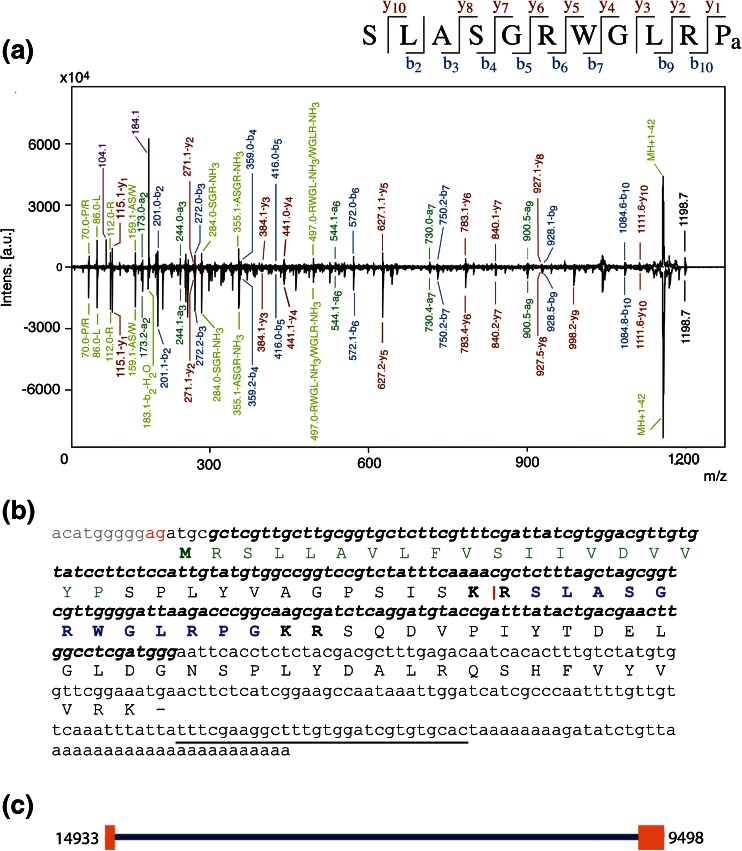
Identification of novel peptide As-NLP-22 in the dorsal and ventral inhibitory motorneurons. **(a- upper spectrum)** MS/MS of the *m/z* 1198.7 peak from a single DI motorneuron. **(a- lower spectrum)** MS/MS of synthetic form of As-NLP-22 reveals nearly identical fragmentation pattern. Peaks representing a- (dark green), b- (blue), y- ions (red), and internal fragments and immonium ions (light green) are labeled. b- and y-ions are summarized in the sequence at the top of the spectrum. Peaks with the *m/z* 104.1 and 184.1 from the fragmentation of a PC head group are seen in MS/MS spectrum of native As-NLP-22, but not that from the synthetic peptide (purple). **(b)** Nucleotide sequence of the *As-nlp-22* transcript, deduced by 5′ and 3′ RACE and confirmed by PCR using unique primers underlined in black (Genbank accession no. KF483662). Green amino acids indicate the signal peptide, with the putative start site in bold. Blue amino acid sequence indicates the encoded peptide, flanked by dibasic cleavage sites in bold. Bolded nucleotide sequence is the portion of the transcript targeted by the *As-nlp-22* riboprobe. Red bases indicate start of exon 1, and the vertical red line shows where two exons are joined together. **(c)** *As-nlp-22* gene model. The gene consists of two exons of 104 and 260 bases, respectively. Exons are separated by an intron 5073 bases in length

The 1198.7 ion was selected for sequencing by tandem MS. De novo interpretation of spectra was done by hand and yielded the sequence S(I/L)ASGRWG(I/L)RPamide (Figure 3a). At this time, the MALDI-TOF/TOF technology is unable to resolve the isoleucine (I)/leucine (L) ambiguity because the two amino acids are isobaric. A BLAST search of the *A. suum* EST library produced the sequence SLASGRWGLRPG (Genbank accession no. BI593877), which is orthologous to the Ce-NLP-22 peptide found in *C. elegans* [44]. Because it is the *A. suum* version, we refer to it as As-NLP-22. The peptide is flanked by dibasic putative cleavage sites and the C-terminal glycine is known to be a substrate for post-translational amidation; thus, the predicted peptide product from the precursor protein is SLASGRWGLRPamide. The MS/MS fragmentation pattern contained nearly the full set of b- and y-ions with an additional set of internal ion fragments. MS/MS spectra of synthetic As-NLP-22 peptide produced a fragmentation pattern nearly identical to that of the native peptide (Figure 3). In MS/MS spectra from both VI and DI neurons, we noticed a single intense peak at *m/z* 1156.7 (MH^+^ – 42 Da). Typically, the loss of 42 Da is due to loss of an acetyl group from a peptide that has been post-translationally acetylated. Knowing this was not a plausible explanation in this case, we hypothesized that the peak was due to the neutral loss of the C-terminal proline side chain (CH_2_CH_2_CH_2_). To test this hypothesis, we performed MS/MS on four additional synthetic peptides with C-terminal prolines, in both the amidated and carboxyl forms, and with variable N-terminal extensions (Online Resource 1). In all cases, an intense peak at MH^+^– 42Da was observed, supporting our hypothesis.

In comparing the MS/MS spectra of natural and synthetic As-NLP-22, there was a striking difference, the presence in the natural peptide spectrum of a strong peak at *m/z* 184.1, and a somewhat less intense peak at *m/z* 104.1 (Figure 3a). Such peaks have been observed previously in MS/MS spectra of peptides from single neurons [23, 41]. In each case, including the present example of As-NLP-22, the MS/MS spectra of synthetic peptides lacked these peaks. Our present hypothesis is that this peak is phosphocholine (PC: [M + H]^+^ 184.073 predicted; 184.069 observed), and that the peak at *m/z* 104.1 is choline. This is supported by the following data. First, the isotopic patterns of this peak are identical with those predicted for PC, and distinct from those of peptides. Second, when synthetic peptide was applied to a MALDI-TOF target plate in the presence of a single dissected neuron (AVK; [23]), MS/MS spectra were obtained both from the zone with pure peptide, and from the region that included the dissected neuron. The results showed that the *m/z* 184.1 and 104.1 peaks only occurred in the region adjacent to the neuron. The differences in the spectra were just like those illustrated in Figure 3, showing the same distinctive peaks in the natural and synthetic peptides. At present, it is unclear why PC is found in MS/MS spectra, since it would be expected that any ions but those chosen from the MS spectrum for fragmentation would be selectively removed. However, the fact that these peaks are routinely found in spectra of natural peptides, but not of their synthetic versions, suggests that this is a common problem. PC is an abundant naturally occurring component of nematode cells since it is used directly as a precursor to phosphatidylcholine [61].

The most likely interpretation of the presence of a peptide with *m/z* 1256.7 is that it is a processing intermediate in the formation of mature As-NLP-22. Its mass is exactly 58 Da more than As-NLP-22, which is explained by the addition of 57 Da from the glycine residue and 1 Da from the mass difference between a C-terminal carboxylic acid and a C-terminal amide. The presence of processing intermediates is not surprising, since the MS analyses are carried out on neuronal cell bodies, where the rough endoplasmic reticulum and the Golgi apparatus, the first sites of processing, are located; processing is completed within the secretory vesicles [29, 62, 63]. C-terminal amidation is the last step in the processing of these peptides, following proteolytic cleavage, trimming by carboxypeptidase and, finally, oxidation of C-terminal glycine residues, leading to the removal of both the carboxyl and α-carbon of glycine, and leaving the amino group as a C-terminal amide. Perhaps it is surprising that these intermediates are not seen more often. In many neurons previously analyzed, no trace of the glycine form of a processed peptide has been detected, although in a few cases, namely the glycine-adduct of AF2 and AF8, the intermediates were detected by MS [42].

A possible explanation of the detection of the glycine adduct in inhibitory motorneurons is that the peptide complement of these cells is very simple, essentially peptide As-NLP-22 and its G adduct; perhaps this simplicity might affect the ionizability of the peptides present during the MALDI process. Unrefined MALDI-TOF MS, such as we have used, is far from being a quantitative technique, and the presence or even the absence of a peptide peak depends not just on the ionizability and concentration of the peptide but also on the complexity of the mixture of peptides present, as manifested in the phenomenon called ion suppression [64]. However, the AF2 and AF8 adducts were detected in much more complex mixtures than that found in the inhibitory motorneurons, making it less likely that suppression is affecting the detectability of G-adducts in general. Perhaps there is an effect of sequence on the efficiency of the amidating enzyme complex, or a difference in the expression levels of enzyme and substrate in different neurons affecting the efficiency of amidation. A thorough analysis of the kinetic properties of the *A. suum* amidating enzyme complex and its activity on different substrates would be useful.

Another example of incomplete processing observed in *A. suum* neurons is incomplete proteolysis, often involving KK cleavage sites [40–42], which apparently are cleaved more slowly than KR or R sites [23, 65]. Proteolytic cleavage is known to be more complex than amidation: in *C. elegans*, there are multiple proteases involved, and the cellular expression of these enzymes is not known. Local sequence around the cleavage sites and the three-dimensional structure of the precursor protein are also possible factors that may affect cleavage specificity and efficiency.

### Cloning of the As-nlp-22 Transcript

To clone the peptide-encoding transcript, we initially used primers specific to SL1, a splice leader sequence on ca. 80% of the known *A. suum* transcripts [66], together with a gene-specific reverse primer, but were unsuccessful. However, the use of 5′ and 3′ RACE with 5′RACE primer *nlp22*-5RACE3 and 3′RACE primer *nlp22-*3RACE allowed us to identify the 5′ and 3′ regions of the transcript, and to identify a 385 base product. To fuse the two RACE product sequences and confirm the validity of the overlapping regions, a full-length PCR reaction was performed using the forward primer *nlp22*-3RACE2 and the reverse primer *nlp22*-5RACE. The deduced amino acid sequence contains an 80 amino acid open reading frame, complete with an initiating methionine N-terminal to a predicted signal peptide, a single copy of the peptide flanked by dibasic cleavage sites, and a 3′ polyA tail (Genbank accession no. KF483662) (Figure 3b). Analysis of genomic sequences showed that the *As-nlp-22* gene is composed of two exons. Exon 1 comprises 104 bases and includes the entire signal peptide region, whereas exon 2 comprises 260 bases and includes the peptide-encoding region. The two exons are separated by an intron of 5073 bases (Figure 3c). There is no evidence that this transcript is trans-spliced to SL1.

### Localization of the As-nlp-22 Transcript by In Situ Hybridization

**Figure 4:**
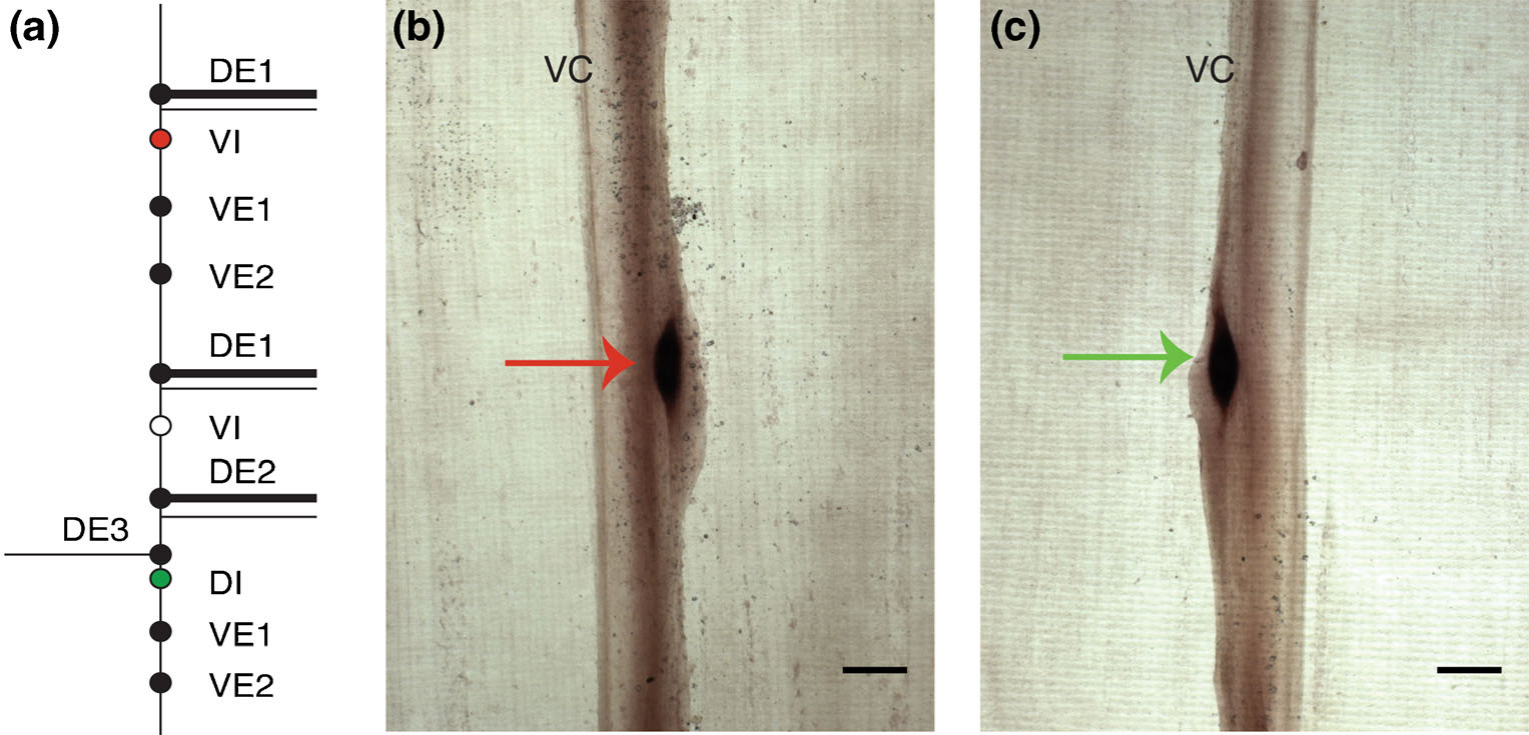
Expression of *As-nlp-22* as determined by ISH. **(a)** Diagram of *A. suum* ventral cord motorneurons in one of the repeating units. Indicated in red (VI) and green (DI) are the cell bodies of neurons shown in b and c. **(b)** VI neuron cell body stained with the *As-nlp-22* riboprobe (red arrow). **(c)** DI neuron cell body stained with the *As-nlp-22* riboprobe (green arrow). Scale bars: 100 μm

To confirm the presence of the *As-nlp-22* mRNA in GABAergic VI and DI ventral cord motorneurons, we used primers *nlp22*-3RACE2 and *nlp22*-5RACE to synthesize a 179 base antisense riboprobe (Figure 3b). As a negative control, a sense strand probe was also synthesized. In the first two to three segments of the 30 worms analyzed, 96% of the DI and VI motorneurons present showed moderate to intense staining when exposed to the antisense probe (Figure 4b, c). No staining was observed in the negative control preparations (data not shown). Interestingly, we also observed a pair of bilaterally symmetrical stained cells in the posterior portion of the ventral ganglion (Online Resource 2a), thought from their position and size to be either AIY or AIM (these neurons are immediate neighbors in the ventral ganglion and have indistinguishable morphology by light microscopy). MS spectra of individual cells dissected from this pair contain peaks at *m/z* 1198.7 and 1256.7, suggesting that they contain authentic As-NLP-22 and the As-NLP-22+G adduct (Online Resource 2b). As in the VI and DI neurons, strong GABA-like immunoreactivity has been detected in one of the AIY/AIM pair [34] but, again, they have not been definitively distinguished from each other. We speculate that GABA and As-NLP-22 co-localize in AIY or AIM as they do in the inhibitory ventral cord motorneurons, but more experiments will be needed to test this. If confirmed, it would strengthen the argument that As-NLP-22 and GABA are often co-expressed, and that As-NLP-22 may be important in the GABA signaling pathway.

### Bioactivity

#### (1) Muscle Strips

**Figure 5:**
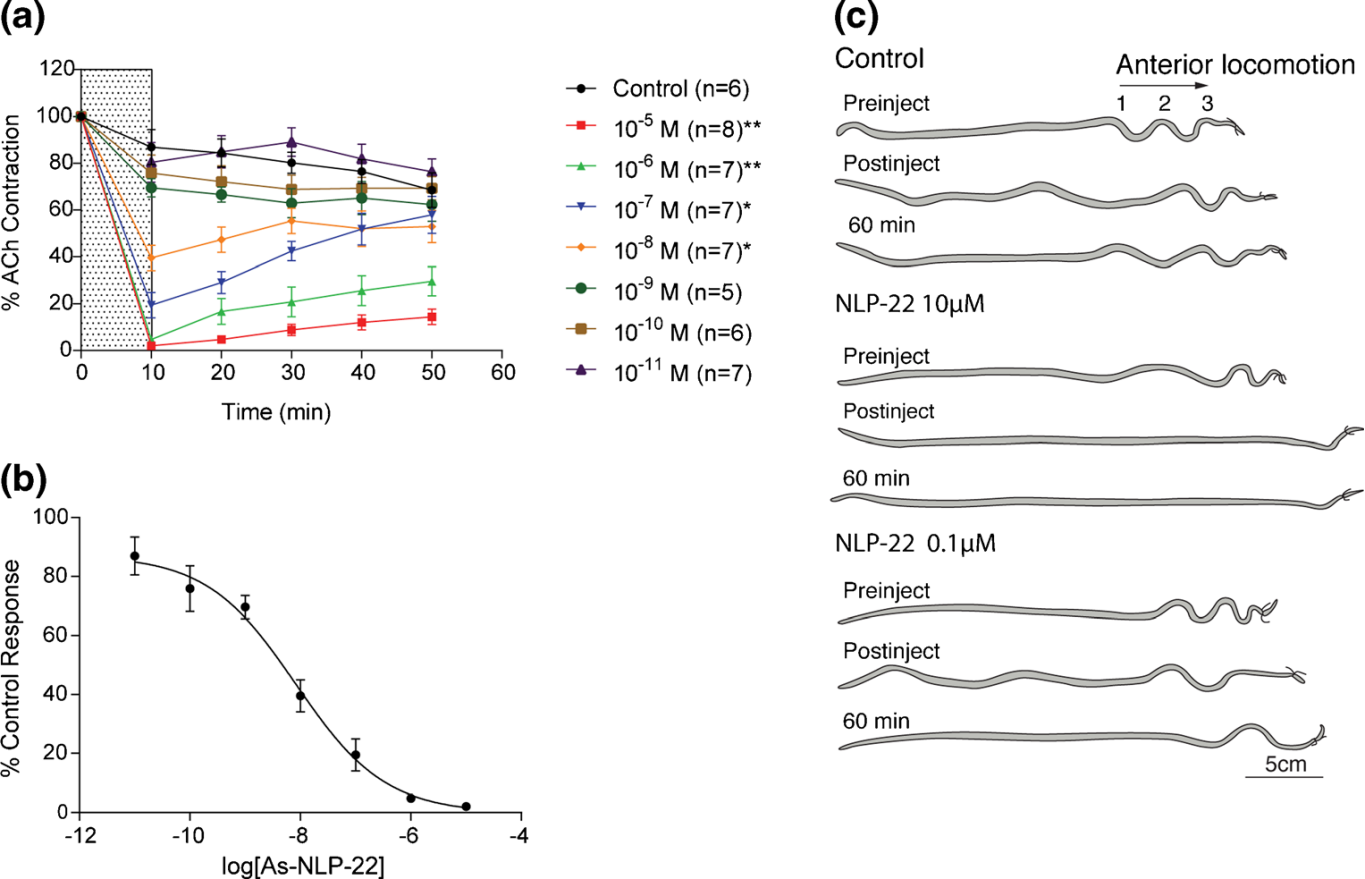
Effects of As-NLP-22 on ACh-induced muscle contraction and gross locomotion. **(a)** Effects of single exposure to synthetic As-NLP-22 on ACh-induced muscle contraction. Peptide was introduced at 0 min and washed out at 10 min, depicted by the shaded region. Strength of contraction is normalized to the initial contraction prior to the application of peptide for each individual worm. Error bars represent standard error of the mean. At concentrations denoted with asterisks, responses were significantly different from control worms at all time points (**P < 0.01, *P < 0.05). **(b)** Dose-response of As-NLP-22. Each data point represents the response in the presence of peptide, expressed as a percentage of the control response. IC_50_ = 8.3 × 10^–9^ M. **(c)** Injection of As-NLP-22 into intact worms causes reduced locomotory behavior. Control worms injected with *Ascaris* saline showed no discernible impairment in locomotory activity (*n* = 13). Worms injected with 10 μM As-NLP-22 displayed paralysis for the duration of the experiment (*n* = 10). Worms injected with 0.1 μM As-NLP-22 (approximates IC_50_ in vivo) displayed a general reduction in locomotory behavior (*n* = 10), though the behavioral responses were more varied than the 10 μM trials

We submitted the peptide As-NLP-22 to functional analysis by measuring its effects on ACh-induced contraction in strips of *A. suum* dorsal muscle, using standard protocols from this laboratory [67, 68]. Exposure to a single dose of 10 μM peptide practically abolishes any contractile response to ACh (2.1% ± 0.4% of the pre-peptide response, n = 8 preparations, P < 0.01). Once the peptide was washed out, a gradual partial recovery (~15%) was observed over 50 min (Figure 5a). Additional concentrations (1 μM, 100, 10, 1, 0.1, 0.01 nM) of As-NLP-22 were tested to determine the IC_50_. A dose-dependent inhibition was observed at all concentrations, followed by a variable extent of recovery once peptide was washed out. A dose–response curve was made by plotting the response in the presence of As-NLP-22 at all seven concentrations as a percentage of the control (Figure 5b). As-NLP-22 exhibits a strong and prolonged inhibitory effect on ACh-induced dorsal muscle strip contraction with an IC_50_ of 8.3 × 10^–9^ M.

We also observed dose-related peptide effects on baseline muscle tension, observed at the end of the 10 min exposure to peptide. The threshold for this effect was between 10^–9^ and 10^–8^ M; the reduction in tension was 1.2 ± 0.2 g, 1.9 ± 0.4 g, 2.2 ± 0.3 g, and 2.6 ± 0.3 g at peptide concentrations of 10^–8^, 10^–7^, 10^–6^, and 10^–5^ M, respectively. After the peptide was washed out, there was no significant reversal of baseline tension to control levels.

#### (2) Behavioral Effects of Injection of Peptides into Intact Worms

To determine the effects of As-NLP-22 on gross locomotory behavior, we injected intact worms with synthetic peptide and observed their behavior in a glass tube with diameter comparable to that of the porcine small intestine, which is the natural habitat of the adult worms. *A. suum* maintain their position in the small intestine by bracing against the walls with pseudo-sinusoidal body waveforms (produced by alternating dorsal and ventral contraction and relaxation of the somatic musculature). These waveforms propagate in the anterior or posterior direction to control forward or backward locomotion, respectively. Anteriorly-propagating waves can be induced by tying surgical thread tightly around the head, producing a stereotypical behavioral response called Head-Restricted Behavior [55, 69, 70]. Peptide solutions can then be injected into the pseudocoelomic cavity posterior to the ligature, and any changes in locomotory behavior recorded.

Injection of 10 μM As-NLP-22 into ligatured worms abolished all locomotory activity posterior to the ligature and decreased body tonus, resulting in flaccid paralysis (*n* = 10; Figure 5c). These worms did not recover appreciably even after 60 min post-injection. This response is in stark contrast to control worms, injected with *Ascaris* saline, which produced normal anteriorly-propagating waves for the duration of the experiment (*n* = 13). Similarly, worms injected with 0.1 μM As-NLP-22 displayed a reduction in the number of anteriorly-propagating waveforms in the body of the worm and in their amplitude, although the results were more variable than the 10 μM trials. Seven of ten worms injected with 0.1 μM As-NLP-22 produced no anteriorly-propagating waves; of these, five exhibited paralysis within 5–10 min post-injection. The remaining 3/10 worms showed no obvious impairment in locomotory behavior. Previous experiments showed that dye solution is diluted ca. 10-fold upon injection into the pseudocoelomic fluid [55], so the peptide concentration resulting from a 0.1 μM As-NLP-22 injection approximates the IC_50_ concentration (8.3 × 10^– 9^ M) deduced from the muscle strip assay. Since the IC_50_ is the concentration that produces 50% of the maximal response, this could account for the variable behavioral responses observed in these trials.

We also made injections of As-NLP-22 into unligatured worms, at concentrations of 10, 1, and 0.1 μM, with similar results (not shown), suggesting that the neurons of the head and nerve ring do not play an important role in the peptide response. In these worms, as in the ligatured worms, no effects on the three-dimensional head-searching movements were observed.

### Comparison with Other Nematodes

It is estimated that the origin of nematode speciation was about 500 million years ago [71]. Nematodes vary dramatically in size and in the ecological niche that each inhabits, yet their basic body plans are highly conserved and the general architecture of nematode nervous systems is very similar. The detailed cellular morphology of neurons has only been determined in two species, *A. suum* and *C. elegans*, which have vastly different life styles: *A. suum* is an intestinal parasite about 30 cm in length, whereas *C. elegans* is a free-living soil nematode, ca. 1 mm in length. The morphology of homologous neurons is virtually identical for both the motorneurons (Figure 1c, d) as well as the other neurons in the nervous system; they differ in size. The expression of classic transmitters in the motorneurons is also highly conserved, with excitors using ACh and inhibitors using GABA.

Structural conservation also extends to neuropeptide sequences. For example, there are four readily identifiable families of peptides, found in 22 other nematodes [72] that share a C-terminal -RPamide sequence. As-NLP-22 belongs to one of these, encoded by the *As-nlp-22* gene. The three other families are encoded by the *nlp-2*, *-23*, and *-46* genes. Confirmation of the peptide sequence of As-NLP-22 and cloning of its peptide-encoding transcript suggests that *A. suum* SLASGRWGLRPamide is the homolog of Ce-NLP-22 and not Ce-NLP-2 as was previously reported [42]. Homologous *nlp-22* genes from other nematodes encode a predicted precursor protein that includes a single copy of the peptide with the characteristic motif S[A/M/L]A[I/N/S]GR[A/M/W][G/Q][M/F/L]RPG, flanked by dibasic or monobasic putative cleavage sites.

**Figure 6:**
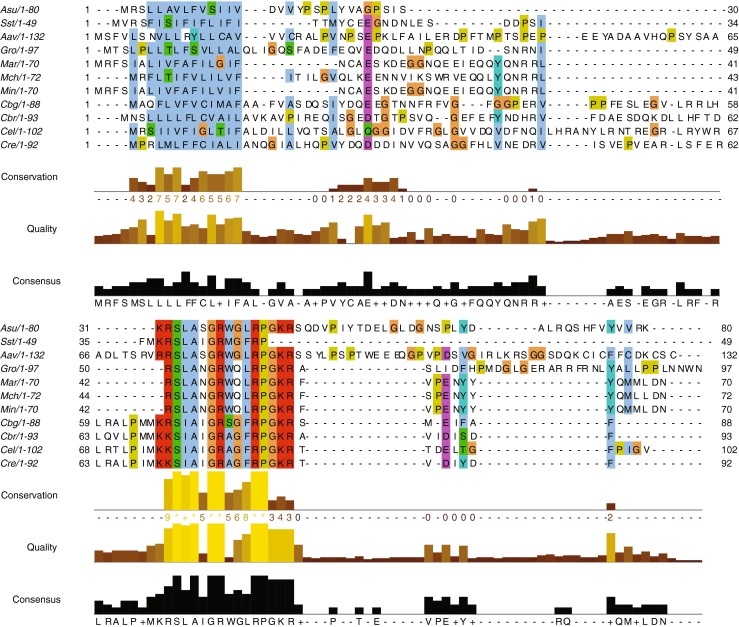
Multiple alignment of nematode *nlp-22* transcripts by MEGA5.1 [56]. Results from 11 nematode species, organized alphabetically by clade, were presented in Jalview [56, 58], and show strong conservation of the predicted encoded peptide and signal peptide. Asu = *Ascaris suum* (Clade III); Sst = *Strongyloides stercoralis* (IVa); Aav = *Aphelenchus avenae* (IVb); Gro = *Globodera rostochiensis* (IVb); Mar =*Meloidogyne arenaria* (IVb); Mch = *Meloidogyne chitwoodi* (IVb); Min = Meloidogyne incognita (IVb); Cbg = *Caenorhabditis briggsae* (V); Cbr = *Caenorhabditis brenneri* (V); Cel = *Caenorhabditis elegans* (V); Cre = *Caenorhabditis remanei* (V)

We conducted BLAST searches of nematode ESTs using both As-NLP-22 and Ce-NLP-22 as queries and identified homologous peptides from 10 other parasitic and free-living nematodes (Online Resource 3), doubling the number of nematode NLP-22 encoding transcripts previously reported by McVeigh et al., 2008 [72]. When sequences were aligned, they fell into two groups differing slightly in their sequence within the conserved nematode NLP-22 motif (Figure 6). Group 1, from clades IVa and V [71, 73], was typified by a C-terminal –FRPamide, whereas group 2, from clades III and IVb, were all from parasitic species and shared a C-terminal –LRPamide sequence. Sequences within members of a group were highly conserved (group 1: SIAIGR[A/S]GFRPamide; group 2: SLA[S/N]GRW[Q/G]LRPamide). The peptide from parasitic nematode *S. stercoralis* is an exception; it appears to be an amalgam of the two, sharing an N-terminal SL- with group 2 and C-terminal –AIGR[X]GFRPamide with group 1. Eventually, it will be interesting to see if these sequence differences are correlated with functional differences, or if they are simply markers of the phylogeny, with shared function.

Little is known about the specific cellular expression of NLP-22 in other nematodes, with the exception of *C. elegans. Ce-nlp-22:GFP* was shown to be expressed in the pair of RIA neurons, head interneurons that regulate a nematode sleep-like state known as lethargus [74]. Over-expression of *Ce-nlp-22* in adult *C. elegans* causes a decrease in locomotion and feeding behavior. Although the structure of *As-nlp-22* and *Ce-nlp-22* are highly similar [72], their reported cellular expression is completely different in the two species, with no expression of *Ce-nlp-22* in inhibitory motorneurons [74].

We have previously suggested that differences in cellular expression of homologous peptides could be at least part of the explanation of how organisms with virtually the same nervous systems can generate different behaviors that enable them to inhabit such different ecological niches. However, a note of caution must be added, since in most cases the cellular localization in *C. elegans* was determined by using GFP constructs that included the promoter and various lengths of 5′ sequence. It is known that sequences controlling gene expression can be remote from the promoter and may also include sequences 3′ to the gene, or intronic regions [75, 76], so it is difficult to know whether the control sequences of the constructs are complete. Corroboratory methods are badly needed. In *A. suum*, we have used in situ hybridization to confirm the localizations observed by MS. Importantly, both methods directly detect the gene products, either the peptides themselves or the peptide-encoding transcript, expressed in the normal worm, without any possible interference in gene expression that could be induced by the introduction of additional gene copies. In addition, in cases where we could raise monospecific antibodies recognizing a single peptide [23], we have used immunocytochemistry as a third technique that also validates the cellular expression. For all three techniques, the large size of *A. suum* neurons is an enormous advantage.

## Conclusions and Future Experiments

This work identifies the first peptide expressed in *A. suum* inhibitory motorneurons. As-NLP-22 is found in the VI and DI inhibitory motorneurons of the ventral cord. We have demonstrated that As-NLP-22 has a potent inhibitory effect on ACh-induced muscle contraction and locomotory behavior. At the moment, the cellular basis for the inhibitory action of As-NLP-22 is unknown. Further investigation of As-NLP-22 by electrophysiological methods, to determine its mechanism of action and postsynaptic partners, will add to the description of the circuitry that controls locomotion and provide valuable information on developing targets for effective drug treatments of nematode parasites. We will also investigate potential interactions between GABA and this peptide, since they are co-expressed in the inhibitory motorneurons.

## Electronic supplementary material

ESM 1 (DOCX 5.90 mb)
