# Supplementary material for: Mass Spectrometry of Single GABAergic Somatic Motorneurons Identifies a Novel Inhibitory Peptide, As-NLP-22, in the Nematode Ascaris suum
Source: J Am Soc Mass Spectrom. 2015 Jul 15;26(12):2009–23. doi: 10.1007/s13361-015-1177-z (PMC4654748; doi:10.1007/s13361-015-1177-z)
Supplement: Supplementary file 1 — (DOCX 5.90 mb) [file 13361_2015_1177_MOESM1_ESM.docx]

**JASMS Online Resource for:**

**Mass Spectrometry of Single GABAergic Somatic Motorneurons Identifies A Novel Inhibitory Peptide, As-NLP-22, in the Nematode *Ascaris suum*.**

Christopher J. Konop^1, *^, Jennifer J. Knickelbine^1,2 *^, Molly S. Sygulla^1^, Colin D. Wruck^1^, Martha M. Vestling^3^, and Antony O. W. Stretton^1,2,4^

^1^Department of Zoology, ^2^ Parasitology and Vector Biology Training Program, ^3^Department of Chemistry, ^4^Neuroscience Training Program,

University of Wisconsin-Madison, Madison Wisconsin, 53706

**Online Resource 1** MS/MS of synthetic peptides with a common C-terminal proline residue similar to As-NLP-22. Peaks representing a (green), b (blue), y (red), and high-intensity internal fragment (purple) ions are labeled, and b and y ions are summarized in the sequence at the top of each spectrum. (a) MS/MS of synthetic As-NLP-23. (b) MS/MS of synthetic As-NLP-46. (c) MS/MS of synthetic As-NLP-2.2. (d) MS/MS of synthetic As-NLP-21.6. Analysis of these spectra identified a common intense ion corresponding to MH+-42 (green)

(a)

(b)

(c)

(d)

**Online Resource 2** Expression of *As-nlp-22* in the ventral ganglion. (a) Pair of cells in the posterior ventral ganglion, stained with the *As-nlp-22* riboprobe. These cells are either the AIY or AIM neurons, which are morphologically indistinguishable in *A. suum*. Scale bar: 100 µm. (b) Mass spectrum from a single AIY or AIM neuron from the ventral ganglion containing peaks with *m/z* 1198.7 and 1256.7

(a)


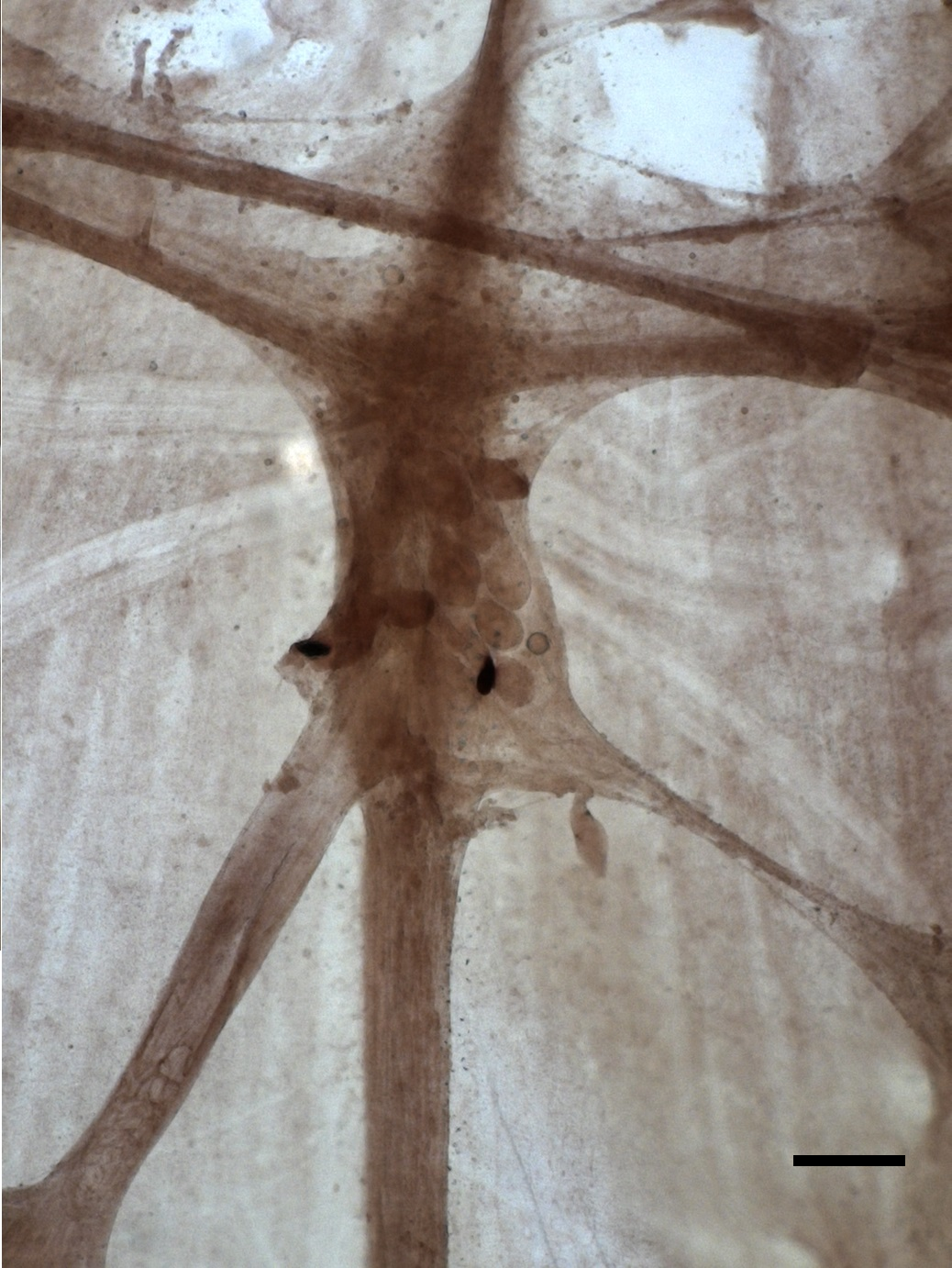


(b)

**Online Resource 3**. Results from BLAST searches of nematode EST databases using both Ce-NLP-22 and As-NLP-22 as queries. In the title line for each sequelog is the three-letter identifier for each species and the GenBank accession number. Green represents a predicted signal peptide (SignalP 4.0) and processed peptides are in blue. Putative basic cleavage residues are indicated in bold

C.e. BLAST results

>Cbg *Caenorhabditis briggsae* CGG79409_1

MAQFLVFVCIMAFAAFVASDQSIYDQEEGTNNFRFVGFGGPERVPPFESLEGVLRRLHLRALPMM**KR**SIAIGRSGFRPG**KR**SMEIFAF

>Cbr *Caenorhabditis brenneri* CBG91628_1

MNSLLLLFLCVAILAVKAVPIREQISGEDTGTPSVQGEFEFYNDHRVFDAESDQKDLLHFTDLQVLPMM**KK**SIAIGRAGFRPG**KR**AVIDISDF

>Cel *Caenorhabditis elegans* CEG82481_1

MRSIIVFIGLTIFALDILLVQTSALGLQGGIDVFRGLGVVDQVDFNQILHRANYLRNTREGRLRYWRLRTLPIM**KK**SIAIGRAGFRPG**KR**TTDELTGFPIGV

>Cre *Caenorhabditis remanei* CRG82931_1

MPRLMLFFCIALIANQGIALHQPVYDQDDDINVVQSAGGFHLVNEDRVISVEPVEARLSFERLRALPIM**KK**SIAIGRAGFRPG**KR**TVDIYDF

>Sst *Strongyloides stercoralis* SSP05227_1

MVRSFISIFIFLIFITTMYCEEGNDNLESDDPSIFM**KR**SLAIGRMGFRP

A.s. BLAST results

>Aav *Aphelenchus avenae* GO481772

MSFVLSNVLLRYLLCAVVVCRALPVNPSEPKLFAILERDPFTMPTSPEPEEYADAAVHQPSYSAAADLTSRV**RR**SLASGRWGLRPG**KR**SSYLPSPTWEEEQGPVPDSVGIRLKRSGGSDQKCICFFCDKCSC

>Asu *Ascaris suum* BI593877

MRSLLAVLFVSIIVDVVYPSPLYVAGPSIS**KR**SLASGRWGLRPG**KR**SQDVPIYTDELGLDGNSPLYDALRQSHFVYVVRK

>Gro *Globodera rostochiensis* EE266657

MTSLPLLTLFSVLLALQLIGQSFADEFEQVEDQDLLNPQQLTIDSNRNI**R**SLANGRWQLRPG**KR**ASLIDFHPMDGLGERARRFRNLYALLPPLNNWN

>Mar *Meloidogyne arenaria* CF358247

MRFSIALIVFAFILGIFNCAESKDEGGNQEEIEQQYQNRRL**R**SLANGRWQLRPG**KR**FVPENYYYQMMLDN

>Mch *Meloidogyne chitwoodi* CB930549

MRFLTIFVLILVFITILGVQLKEENNVIKSWRVEQQLYQNRRL**R**SLANGRWQLRPG**KR**FSPENYYYQMLLDN

>Min *Meloidogyne incognita* AW783047

MRFSIALIVFAFILVIFNCAESKDEGGNQEEIEQQYQNRRL**R**SLANGRWQLRPG**KR**FVPENYYYQMMLDN
